# Dolutegravir‐induced acquired sideroblastic anemia in a HIV positive patient: A challenging hematologic complication

**DOI:** 10.1002/ccr3.8301

**Published:** 2023-12-09

**Authors:** Kiran Dhonju, Ashmita Gautam, Abhinav Dahal, Niraj Kumar Sharma, Divas Adhikari, Lina Devkota, Prabhat Adhikari, Sampurna Tuladhar, Bishnu Deep Pathak, Sabin Banmala

**Affiliations:** ^1^ Sukraraj Tropical and Infectious Disease Hospital Kathmandu Nepal; ^2^ Tribhuvan University Institute of Medicine Kathmandu Nepal; ^3^ Bharatpur Hospital Bharatpur Nepal; ^4^ Center for American Medical Specialists Nepal; ^5^ Civil Service Hospital Kathmandu Nepal; ^6^ Nepalese Army Institute of Health Sciences Kathmandu Nepal

**Keywords:** acquired sideroblastic anemia, adverse drug reaction, Dolutegravir, HIV, Naranjo scale

## Abstract

Dolutegravir, the most recent antiretroviral drug with high efficacy, good tolerability, infrequent drug–drug interactions, and a favorable safety profile has not been reported in current literature as a cause of acquired sideroblastic anemia. Here, we present a 35‐year‐old male patient who was diagnosed with acquired sideroblastic anemia to Dolutegravir therapy.

## Graphical abstract

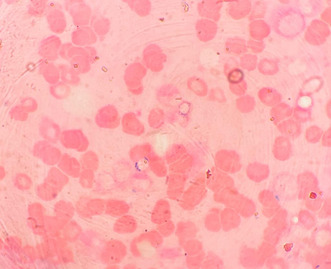

## 1 INTRODUCTION

Sideroblastic anemias (SAs) encompass a group of bone marrow disorders characterized by the abnormal accumulation of iron in the mitochondria of erythroid precursors. This leads to the formation of ring sideroblasts, where iron‐laden mitochondria surround the nuclei of erythroblasts.^1^ Ring sideroblasts can be present in various pathological conditions, including both congenital and acquired forms of sideroblastic anemias.^2^ The underlying mechanisms leading to different forms of sideroblastic anemias are diverse. However, in all cases, the abnormal accumulation of iron is attributed to disruptions in mitochondrial proteins involved in regulating heme synthesis or Fe/S cluster synthesis. Additionally, impairment in translating mitochondrially encoded proteins contributes to this iron deposition. Consequently, these alterations give rise to ineffective erythropoiesis and iron accumulation in tissues, leading to tissue iron overload.^1^ It has been associated with antituberculin agents, chloramphenicol, and ethanol. While several etiological factors have been implicated in acquired sideroblastic anemia, including genetic mutations and exposure to toxins,^3^ the association between antiretroviral therapy and the development of acquired sideroblastic anemia (ASA) is rare and has not been reported to date. Here we report the development of sideroblastic anemia in a patient who was on Dolutegravir therapy. Dolutegravir, an integrase strand transfer inhibitor (INSTI), exerts its antiviral activity by binding to the active site of HIV integrase, preventing the stand‐transfer step crucial in the retroviral DNA integration process, thus reducing viral load and slowing down disease progression.^4^ The association of ASA with Dolutegravir has not been established in the current literature. Naranjo method for estimating the probability of adverse drug reaction (ADR) was used to show the casual association.^5^

## 2 CASE PRESENTATION

A 35‐year‐old male from Boudha, Kathmandu, was diagnosed with HIV on September 14, 2016. As a result, his first antiretroviral therapy (ART) clinic visit occurred on March 13, 2017, and his CD4 count was measured at 500 cells/mm^3^. He started ART on March 16, 2017 with the Tenofovir Disoproxil Fumarate (TDF), Lamivudine (3TC), and Efavirenz (EFV) regimens.

During the initial days of treatment, he developed a rash as a side effect of Efavirenz (EFV). To manage the rash, cortilone (corticosteroid) was prescribed for a week, and the symptoms subsided. He underwent a complete blood count (CBC), renal function tests (RFT), and liver function tests (LFT) as part of his initial follow‐up on March 29, 2017. The results showed a WBC count of 5400 cells/mm^3^, Hb of 14.30 g/dL, platelet count of 21,800 cells/mm^3^, CD4 count of 525 cells/mm^3^, creatinine (Cr) of 1.0 mg/dL, and ALT (SGPT) of 57 U/L.

Every 6 months, viral load testing was carried out, and the initial result on September 20, 2017, showed less than 20 copies, demonstrating effective viral suppression. He was started on Isoniazid Preventive Therapy on August 24, 2018 to prevent opportunistic tuberculosis infection. After 6 months of unremarkable therapy, it was discontinued.

Efavirenz is known to be associated with neuropsychiatric symptoms like delirium, anxiety, acute psychosis, and an increased risk of suicidal thoughts. Following the national protocol guidelines, EFV was replaced with another drug, that is Dolutegravir to avoid potential side effects on June 10, 2020.

**FIGURE 1:**
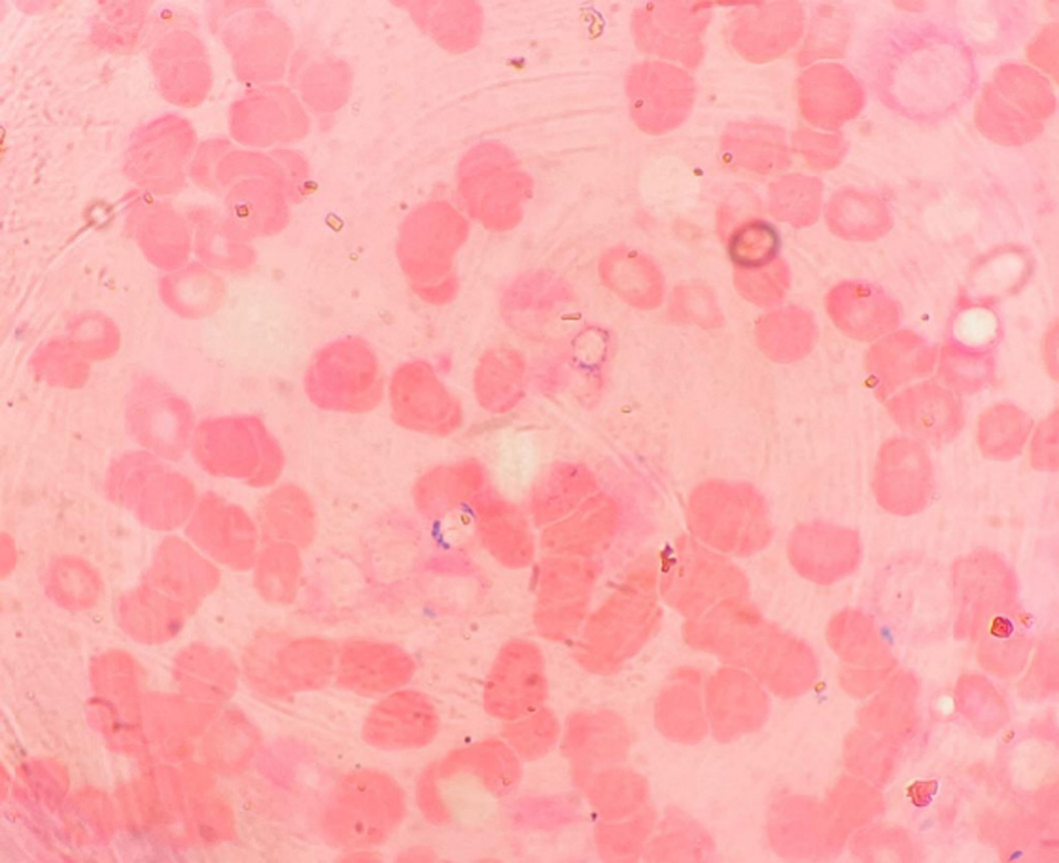
Bone marrow aspirate shows myelodysplastic syndrome with ring sideroblasts.

**FIGURE 2:**
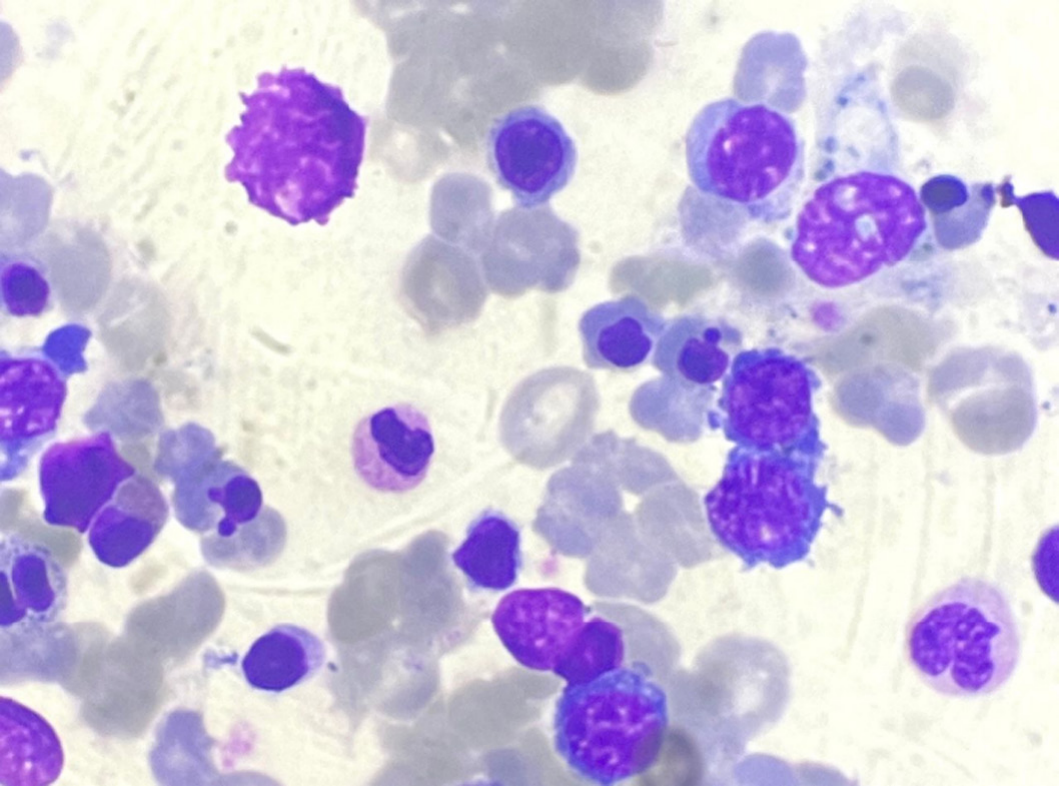
Bone marrow biopsy shows mildly hypercellular marrow with erythroid hyperplasia.

After 2 months of continuing the TDF, 3TC, and DTG regimens, the patient came to OPD complaining of generalized fatiguability, dizziness, and headaches for 1 week. Upon admission, the patient exhibited slight pallor without hepatosplenomegaly or lymphadenopathy. CBC revealed Hb 4.5 gm/dL, MCV of 68.6 fL, and MCH of 23.6 pg. Peripheral blood smear (PBS) showed microcytic hypochromic red cells. Pencil cells were also seen. Any atypical cells/hemoparasites were not present. The stool for occult blood was negative. A blood transfusion was done, and Hb was raised to 7.7gm/dL. Iron deficiency anemia was suspected, and the patient was prescribed iron tablets. On follow‐up after a week, the patient complained the symptoms did not subside. Hb was reduced to 3.6 gm/dL. Hematocrit was 9.9% and MCV of 80 fL. PBS revealed hypochromasia with anisopoikilocytosis with few polychromatic cells, smudge cells, and atypical white blood cells. LDH was 1600 U/L with total bilirubin and direct bilirubin of 2.7 mg/dL and 0.9 mg/dL, respectively. The iron profile showed serum iron of 182 μg/dL, TIBC of 251 μg/dL, %Transferrin saturation of 72.5%, and serum ferritin of 1950 ng/mL. Antinuclear antibodies were 4.8 ODI (negative), and double‐stranded DNA was 6.0 IU/ML (negative). The direct Coombs test was negative. The blast cell morphology and immunophenotyping are typically used to diagnose hematological disorders and malignancies. Chromosomal analysis aids in diagnosis; however, chromosomal disorders are not a factor in all hematological malignancies. Myelodysplastic syndrome with ring sideroblasts (>15%) was seen in bone marrow aspirates as shown in Figure 1. Mildly hypercellular marrow with erythroid hyperplasia was present in bone marrow biopsy as shown in Figure 2. The combination of these findings, along with the presence of marked myelofibrosis on bone marrow biopsy, led to a suspected diagnosis of idiopathic acquired sideroblastic anemia with a pre‐leukemic state. According to Naranjo's Adverse Drug Reaction (ADR) Assessment Scale, it received the “probable” level rating after scoring 5 points, as shown in Table 1.

**TABLE 1:** Naranjo ADR probability score.

S.N.	Questions	Yes	No	Do not know
1.	Are there previous conclusive reports on this reaction?	+1	0	**0**
2.	Did the adverse event appear after the suspected drug was administered?	**+2**	−1	0
3.	Did the adverse event improve when the drug was discontinued or a specific antagonist was administered?	**+1**	0	0
4.	Did the adverse event reappear when the drug was re‐administered?	+2	−1	**0**
5.	Are there alternate causes, other than the drug, that could solely have caused the reaction?	−1	**+2**	0
6.	Did the reaction reappear when a placebo was given?	−1	+1	**0**
7.	Was the drug detected in the blood (or other fluids) in a concentration known to be toxic?	+1	0	**0**
8.	Was the reaction more severe when the dose was increased or less severe when the dose was decreased?	+1	0	**0**
9.	Did the patient have a similar reaction to the same or similar drugs in any previous exposure? Was the adverse event confirmed by objective evidence?	+1	**0**	0
10.	Was the adverse event confirmed by objective evidence?	+1	0	**0**

*Note*: Bold values denote scoring for our case. Total score = +5. Score ≥9 = Definitive. Score 5–8 = probable. Score ≤0 = doubtful.

As a result, Dolutegravir was replaced with Raltegravir and the new ART regimen was started.

Follow‐up laboratory investigations conducted 1 month after discontinuing Dolutegravir are compared with those conducted before discontinuing the drug as shown in Table 2. The patient's symptoms of fatigue and weakness also improved significantly. Subsequent monitoring over a period of 6 months demonstrated sustained hematologic recovery without the recurrence of anemia.

**TABLE 2:** Comparison of blood parameters before and after discontinuation of Dolutegravir therapy.

PARAMETERS	Before discontinuation of Dolutegravir therapy	After discontinuation of Dolutegravir therapy	UNIT	Reference range
Hemoglobin level	5.3	13.5	g/dL	13.5–17.5
Platelet count	435,000	250,000	Cells/cumm	150,000–400,000
Leukocyte count	3400	5500	Cell/cumm	4000–11,000
Myeloblasts	8	1	%	≤5
Myelocytes	10	3	%	1–10
Neutrophils	22	50	%	40–70
Lymphocytes	45	30	%	20–40
Monocytes	2	5	%	2–10
Eosinophils	5	2	%	1–6
Serum iron levels	182	65	μg/dL	65–175
Total iron‐binding capacity	107	270	μg/dL	250–400
Ferritin levels	>1500	50	ng/mL	20–250
Lactate dehydrogenase (LDH)	1600	145	U/L	45–200
Calcium level	8.2	8.5	mg/dL	8.4–10.2

## 3 DISCUSSION

With the features like high efficacy, good tolerability, a favorable safety profile, and the absence of significant drug–drug interaction, Integrase inhibitors are now recommended as the preferred first‐line regimen for people living with HIV initiating antiretroviral therapy.^6^, ^7^ They are a class of antiretroviral medications that act by blocking the integrase enzyme responsible for integrating double‐stranded DNA into the host cell genome, thus inhibiting HIV from multiplying and infecting other cells.^8^

Dolutegravir, which was approved in 2013, is the most recent integrase inhibitor that is equally effective as or better than current treatment regimens in both treatment‐experienced and treatment‐naive individuals.^9^ Common adverse effects include headache, nausea, and diarrhea. With Dolutegravir, there is no increase in triglycerides, low‐density lipoprotein, or total cholesterol that is seen with protease inhibitors.^10^

In our case, the patient developed the features of anemia like fatigue, generalized weakness, and shortness of breath after taking Dolutegravir (DTG) along with Tenofovir disoproxil fumarate (TDF) and Lamivudine (3TC) for 2 months. Initially, iron deficiency anemia was suspected, so baseline investigations along with iron profile were sent. But the serum ferritin level was high. Peripheral blood smear, anti‐nuclear antibody test, direct Coomb's test, and chromosomal analysis were also done. Considering the possibility of sideroblastic anemia, bone marrow aspirate and biopsy were planned. Sideroblastic anemias include a heterogeneous group of inherited/congenital and acquired disorders characterized by ineffective erythropoiesis and the presence of ring sideroblasts in the bone marrow aspirate. Ring sideroblasts, aberrant erythroblasts with iron‐loaded mitochondria, are seen as a perinuclear ring of green‐blue granules when stained with Prussian blue.^2^ There should be a minimum of five granules encircling one‐third of the nuclear diameter in this Prussian blue staining process known as the Perls reaction.^11^ Sideroblastic anemias can be grouped into inherited and acquired forms. The inherited forms are rare and characterized by three modes of inheritance: X‐linked (XLSA), autosomal recessive (ARSA), or maternal. Acquired sideroblastic anemias can be classified into clonal (neoplastic) and nonclonal (nonneoplastic).^12^, ^13^ Nonclonal, acquired sideroblastic anemias can be encountered in the setting of alcohol addiction and associated pyridoxine deficiency and are typically caused by dietary deficiencies or ingestion/accumulation of toxins.^14^ Alternative reasons include copper deficiency^15^ and medications including chloramphenicol, linezolid, penicillamine, busulfan, melphalan, and cycloserine (due to pyridoxine deficiency).^11^, ^16^, ^17^

Although the prevalence of sideroblastic anemia has not been defined, it is rare.^12^ Regardless of the genetic variety and pathophysiologic variation among sideroblastic anemia subtypes, the diagnostic approach for sideroblastic anemia adheres to the same fundamental ideas. Before doing a clinical and genetic examination for congenital sideroblastic anemia, reversible causes of sideroblastic anemia and myelodysplastic syndrome must be ruled out. By definition, iron staining in the bone marrow aspirate is necessary for the diagnosis of all kinds of sideroblastic anemia. Excessive alcohol use, heavy metal toxicity, isoniazid, chloramphenicol, or linezolid treatment, malnutrition with following copper, vitamin B6, or thiamine deficiencies, as observed after bariatric surgery, are all reversible factors that should be carefully examined while eliciting a history.^12^ When reversible causes of sideroblastic anemia are considered, one must take into account atypical connections that patients might not be aware of. For example, prolonged high‐zinc consumption from fad diets or routine use of zinc‐containing denture treatments may not be evident causes of zinc poisoning and hemolytic anemia. This justifies including copper, ceruloplasmin, zinc, and lead levels in the diagnostic assessment even in situations where there is not a clear connection. In our patient, we ruled out these reversible causes by taking a detailed medical history, family history, personal history, and physical examination. During the first 3 years of continuing the Tenofovir Disoproxil Fumarate (TDF), Lamivudine (3TC), and Efavirenz (EFV) regimen, the patient never complained of symptoms of anemia like generalized fatigability, shortness of breath, weakness, etc. But after 2 months of introduction of Dolutegravir into the ART regimen, the symptoms of anemia started.

Whatever reversible cause might be causing sideroblastic anemia in this patient, removal of the offending agent is the first step of its management. In our case, the patient's condition improved after stopping Dolutegravir for a month. During a later six‐month monitoring period, there was a sustained hematologic improvement without a recurrence of anemia. This discontinuation, along with the clinical presentation and bone marrow aspirate findings suggested Dolutegravir as the reversible cause of acquired sideroblastic anemia.

## 4 CONCLUSION

To our knowledge, this is the first case report about Dolutegravir causing acquired sideroblastic anemia. Even though Dolutegravir is a new antiretroviral drug with effective virologic suppression, good tolerability, infrequent drug–drug interactions, and once‐daily administration, patients might be experiencing certain side effects like sideroblastic anemia. Early recognition and prompt management of such issues are a must since they might impact drug compliance and ultimately the quality of life. The majority of individuals with anemia who are treated in primary care clinics are thought to have iron deficiency anemia, which is more frequent. Clinicians must, however, be aware of the potential for sideroblastic anemia. To properly manage the patient, the primary care physician, nurse practitioner, and internist must be able to identify and diagnose this illness. To assess the prognosis and epidemiology of this condition, more clinical research is also required.

## AUTHOR CONTRIBUTIONS

**Kiran Dhonju:** Conceptualization; data curation; investigation; methodology; resources; supervision; writing – original draft; writing – review and editing. **Ashmita Gautam:** Data curation; investigation; methodology; project administration; resources; writing – original draft. **Abhinav Dahal:** Conceptualization; data curation; investigation; resources; supervision; writing – original draft; writing – review and editing. **Niraj Kumar Sharma:** Investigation; resources; supervision; writing – review and editing. **Divas Adhikari:** Supervision; writing – original draft; writing – review and editing. **Lina Devkota:** Investigation; resources; writing – review and editing. **Prabhat Adhikari:** Supervision; writing – review and editing. **Sampurna Tuladhar:** Resources; writing – review and editing. **Bishnu Deep Pathak:** Supervision; writing – review and editing. **Sabin Banmala:** Writing – review and editing.

## FUNDING INFORMATION

None.

## CONFLICT OF INTEREST STATEMENT

The authors report no conflicts of interest.

## CONSENT

Written informed consent was obtained from the patient to publish this report in accordance with the journal's patient consent policy.

## Data Availability

No data were used.
